# Procalcitonin Levels Associate with Severity of *Clostridium difficile* Infection

**DOI:** 10.1371/journal.pone.0058265

**Published:** 2013-03-07

**Authors:** Krishna Rao, Seth T. Walk, Dejan Micic, Elizabeth Chenoweth, Lili Deng, Andrzej T. Galecki, Ruchika Jain, Itishree Trivedi, Marie Yu, Kavitha Santhosh, Cathrin Ring, Vincent B. Young, Gary B. Huffnagle, David M. Aronoff

**Affiliations:** 1 Department of Internal Medicine, University of Michigan Health System, Ann Arbor, Michigan, United States of America; 2 Division of Infectious Diseases, University of Michigan Health System, Ann Arbor, Michigan, United States of America; 3 Division of Geriatric Medicine, University of Michigan Health System, Ann Arbor, Michigan, United States of America; 4 Division of Pulmonary and Critical Care, University of Michigan Health System, Ann Arbor, Michigan, United States of America; 5 Department of Biostatistics, University of Michigan Health System, Ann Arbor, Michigan, United States of America; 6 Department of Microbiology and Immunology, University of Michigan Health System, Ann Arbor, Michigan, United States of America; 7 Department of Pharmacy Services, University of Michigan Health System, Ann Arbor, Michigan, United States of America; 8 University of Michigan Medical School, Ann Arbor, Michigan, United States of America; Institute Pasteur, France

## Abstract

**Objective:**

*Clostridium difficile* infection (CDI) is a major cause of morbidity and biomarkers that predict severity of illness are needed. Procalcitonin (PCT), a serum biomarker with specificity for bacterial infections, has been little studied in CDI. We hypothesized that PCT associated with CDI severity.

**Design:**

Serum PCT levels were measured for 69 cases of CDI. Chart review was performed to evaluate the presence of severity markers and concurrent acute bacterial infection (CABI). We defined the binary variables *clinical score* as having fever (T >38°C), acute organ dysfunction (AOD), and/or WBC >15,000 cells/mm^3^ and *expanded score*, which included the *clinical score* plus the following: ICU admission, no response to therapy, colectomy, and/or death.

**Results:**

In univariate analysis log_10_ PCT associated with *clinical score* (OR 3.13, 95% CI 1.69–5.81, *P*<.001) and *expanded score* (OR 3.33, 95% CI 1.77–6.23, *P*<.001). In a multivariable model including the covariates log_10_ PCT, enzyme immunoassay for toxin A/B, ribotype 027, age, weighted Charlson-Deyo comorbidity index, CABI, and extended care facility residence, log_10_ PCT associated with *clinical score* (OR 3.09, 95% CI 1.5–6.35, *P* = .002) and *expanded score* (OR 3.06, 95% CI 1.49–6.26, *P* = .002). PCT >0.2 ng/mL was 81% sensitive/73% specific for a positive *clinical score* and had a negative predictive value of 90%.

**Conclusion:**

An elevated PCT level associated with the presence of CDI severity markers and CDI was unlikely to be severe with a serum PCT level below 0.2 ng/mL. The extent to which PCT changes during CDI therapy or predicts recurrent CDI remains to be quantified.

## Introduction

The clinical manifestations of *Clostridium difficile* infection (CDI) range from asymptomatic carriage, to acute, self-limited diarrheal illness, to fulminant and sometimes fatal pseudomembranous colitis [1], [2]. Recently, CDI has become a major cause of morbidity and mortality in hospitalized patients [3], [4], [5], [6], [7]. Although it is known that toxin-production by the pathogen is essential in the pathophysiology of CDI, it is unclear what causes some patients to experience severe disease. Furthermore, if potentially severe cases of CDI can be identified on the basis of serologic markers, it may be possible to improve clinical outcomes [8]. Thus, new studies of potentially useful biomarkers are needed in CDI.

Studying a biomarker as an indicator of disease severity has been limited in part by a lack of a consensus definition of severe CDI. McDonald et al. [9] endorsed a surveillance definition of severe CDI that focused on outcomes attributable to CDI, including any of the following criteria within 30 days: admission to an intensive care unit (ICU); the need for surgery (such as colectomy); and death. The Society for Healthcare Epidemiology of America and the Infectious Diseases Society of America 2010 clinical practice guidelines [10] recommended using clinical criteria that may be present at the time of diagnosis: leukocytosis (white blood cell count ≥15,000 cells/mm^3^), 1.5 fold rise in serum creatinine, hypotension/shock, ileus, or megacolon. A systematic review by Belmares et al. [11] found seven sets of criteria in published studies to measure the severity of CDI or predict outcome of treatment, using various combinations of 17 clinical variables including diarrhea frequency, leukocytosis, fever, hypotension, and renal insufficiency. There is little consensus in the field as to which of these factors, or combinations of these factors, may be the most clinically relevant.

Numerous risk factors that increase the risk of severe CDI have been identified including age [12], decreased functional status [13], and comorbid illness [14], [15], [16]. At least some of these risk factors, however, are subjective. A completely objective biomarker of CDI severity would have the following characteristics: it is i) specifically prognostic, ii) easily acquired from patients, iii) easily quantified, and iv) minimally affected by other patient co-morbidities. Procalcitonin (PCT) is a biomarker that has shown specificity for bacterial infection and its utility has been demonstrated when used to guide antibiotic therapy in clinical algorithms for respiratory infections [17], sepsis [18], [19], postoperative infections [20], [21], and ventilator-associated pneumonia [22]. Though it has been shown to have some ability to differentiate bacterial enterocolitis from other causes of diarrhea [23], [24], its role as a marker for severe CDI has not yet, to our knowledge, been systematically investigated. Our objective was to evaluate if an association exists between elevated PCT and CDI severity.

## Materials and Methods

### Ethics Statement

This study was approved by the University of Michigan Institutional Review Board. Written informed consent for participation in this study was obtained from all patients.

### Setting

The University of Michigan Health System (UMHS) has a 930-bed, tertiary care inpatient facility. The institution utilizes an electronic medical record system providing access to patient records.

### Patient Selection

Inpatients ≥18 years of age with diarrhea were consented and consecutively enrolled in the study after having a stool sample test positive for the presence of toxigenic *C. difficile*. Initial sample testing was performed at the discretion of the inpatient care team and samples were sent to the Clinical Microbiology Laboratory in Cary-Blair transport medium. Testing was performed on stools using the C. DIFF QUIK CHEK COMPLETE® test (Techlab, Inc., Blacksburg, VA) for *C. difficile* glutamate dehydrogenase (GDH) and toxins A or B by enzyme immunoassay (EIA). All GDH^+^/toxin^−^ stool tests were subjected to analysis for the *tcdB* gene by real-time PCR (BD GeneOhm™ Cdiff Assay; Franklin Lakes, New Jersey). Attempts to confirm positive *C. difficile* tests were made by anaerobic culture on taurocholate-cycloserine-cefoxitin-fructose agar at 37°C and PCR, and strains were ribotyped using high-throughput, fluorescent PCR ribotyping as described elsewhere [25]. We enrolled two separate cohorts from October 25, 2010 to March 29, 2011 and October 31, 2011 to April 4, 2012 and all were selected based on the availability of serum for testing. Patients who were pregnant or did not have diarrhea were excluded.

### PCT Measurement

Serum PCT was measured using the VIDAS® BRAHMS PCT assay (bioMérieux, Inc., Durham, NC), an FDA-approved automated immunofluorescent assay. Reagents were generously provided by bioMérieux. Serum was collected on the date of enrollment, which was <24–72 hours after a positive toxigenic *C. difficile* stool test (median <24 hours) and stored at −80°C until analysis.

### Clinical Epidemiology

We conducted retrospective chart reviews with multiple reviewers, blinded to the PCT results, to assess the presence of clinical parameters including immunosuppression (innate or exogenous); concurrent acute bacterial illness (CABI); acute organ dysfunction (AOD); lack of response to initial therapy (defined as a need to switch initial therapy or a lack of clinical response within 5 days); intensive care unit (ICU) stay, colectomy, or death attributable to CDI within 30 days; residence in an extended care facility (ECF) upon admission and discharge; early relapse (recurrent symptoms <2 weeks after diagnosis); and any recurrence. Recurrence consisted of any positive assay in our hospital system >14 days from the index case. AOD was defined as acute kidney injury per RIFLE criteria [26], acute respiratory distress syndrome (ARDS) (if listed in the chart or meeting criteria of a PaO_2_/FiO_2_<200 and diffuse pulmonary infiltrates with acute onset), new/worse heart failure (if listed in the chart), liver failure (new/worse coagulopathy or hepatic encephalopathy), and/or shock (systolic blood pressure <90 mm Hg or need for pressors/inotropes). We assigned published CDI surveillance definitions [9] to each patient (hospital onset, healthcare facility acquired, HOHA; community onset, healthcare facility acquired, COHA; community acquired, CA; or indeterminate, IND). Two reviewers independently extracted data from patient charts and differences were resolved by a third independent reviewer.

Data readily available in the electronic medical record via structured query were also collected including age, gender, inpatient medications, vital signs, and laboratory values - white blood cell (WBC) count and albumin, within 24 hours of CDI diagnosis. Interviews were conducted with the patients or their families upon enrollment to obtain information on number of stools per day and baseline functional status. We asked about the ability to perform activities of daily living (ADLs) including bathing, transferring, walking, dressing, grooming, and feeding independently or with assistance and scored patients from zero to six with one point assigned for each activity requiring assistance. Comorbid conditions were assessed through structured query for ICD-9 codes as described by Deyo et al. [27] Weights from the original Charlson comorbidity index [28] were assigned to the individual variables in the Deyo modification to create a weighted Charlson-Deyo comorbidity index. Because ICD-10 codes were unavailable, the presence of renal disease and a history of cancer were defined using the Deyo modification of the Charlson comorbidity index and combined with age to create a modified ARC comorbidity score [16].

### Data Analysis

To test the hypothesis that PCT elevation is associated with CDI severity, our primary outcome of interest, we defined two dependent binary variables using the following CDI severity markers: WBC >15,000 cells/mm^3^; fever (temperature >38°C); AOD; no initial response to therapy; ≥seven stools per day; and ICU stay, colectomy, or death attributable to CDI within 30 days. The first variable: the *clinical score* (including data available at the time of diagnosis) was true if WBC >15,000 cells/mm^3^, fever, and/or AOD was present and the second variable, the *expanded score* (incorporating outcome measures), was true if the *clinical score* was true or no initial response to therapy, ICU stay, colectomy, and/or death was present. Statistical analysis was conducted using the programs R 2.15.0 (http://www.r-project.org), SAS 9.3 (SAS Institute, Inc., Cary, NC), and Graphpad Prism 5.04 (Graphpad Software, Inc., La Jolla, CA). Given the broad range of PCT values in our patients, we assigned undetectable PCT levels a value of 0.01 and performed log transformation on the PCT data prior to analysis. We used univariate logistic regression to test if log_10_ PCT was significantly associated with a positive *clinical score* or *expanded score*. We used univariate logistic regression with the following additional risk factors to see if they were associated with a positive *clinical score* or *expanded score*: age, gender, ADL score, immunosuppression, renal disease, ECF residence, modified ARC score, weighted Charlson-Deyo comorbidity score, CABI, CDI treatment for >48 hrs at time of PCT measurement, and CDI surveillance definitions (CA, COHA, and HOHA). The amount of time between CDI diagnosis and PCT measurement varied. We assessed the association between this timing and both PCT level and CDI severity using linear and logistic regression, respectively. We used multiple logistic regression with the above risk factors to examine how they interacted with log_10_ PCT in a pairwise fashion. The final multivariable model for log_10_ PCT and the scoring systems included age, weighted Charlson-Deyo comorbidity score, CABI, and ECF residence and the inclusion of these factors was based on clinical importance and/or a *P*<0.05 on univariate analysis.

As a large number of patients had ≥seven stools per day we looked at this as a secondary outcome separately for an association with log_10_ PCT and also tested how it affected the results when included with the variables in the *clinical score* as a binary variable. We also tested whether log_10_ PCT was associated with secondary outcomes such as length of stay, need for an increased level of care upon discharge, or early relapse. To evaluate the performance of PCT as a diagnostic test for CDI severity, receiver-operator characteristic (ROC) curves were used to set the optimal cutoff values. An association between PCT and recurrent disease was tested using Kaplan-Meier survival analysis with the two patient groups stratified by an optimal PCT cutoff value determined by ROC curves; significance testing was done using the Chi-Square test.

## Results

### Patient Characteristics

**Figure 1:**
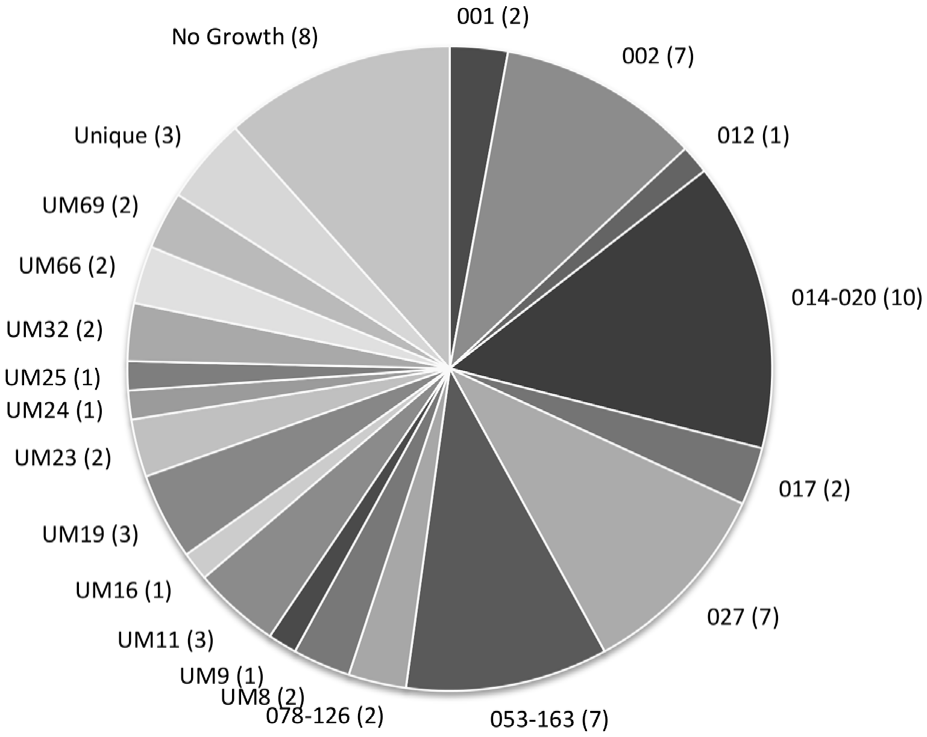
Illustration of *Clostridium difficile* isolates by ribotype. This chart shows a diverse population of *Clostridium difficile* isolates in our cohort. Twenty-two different ribotypes were identified. The prefix “UM” is used for ribotypes that were different from known reference isolates and were identified at the University of Michigan Health System (UMHS) more than once. The “Unique” grouping is for isolates that were identified only once at UMHS. *Labels*: Ribotype (number of patients).

**Figure 2:**
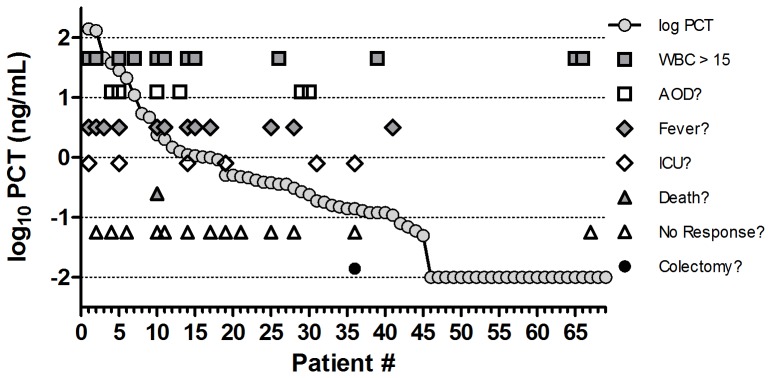
Procalcitonin (PCT) levels and presence of severity markers. This graph shows that patients with elevated PCT levels have an abundance of severity markers, while those with low or undetectable PCT levels have few. Patients are ordered by decreasing log_10_ PCT value. (*WBC*: white blood cell; *AOD*: acute organ dysfunction; *ICU*: intensive care unit).

**Figure 3:**
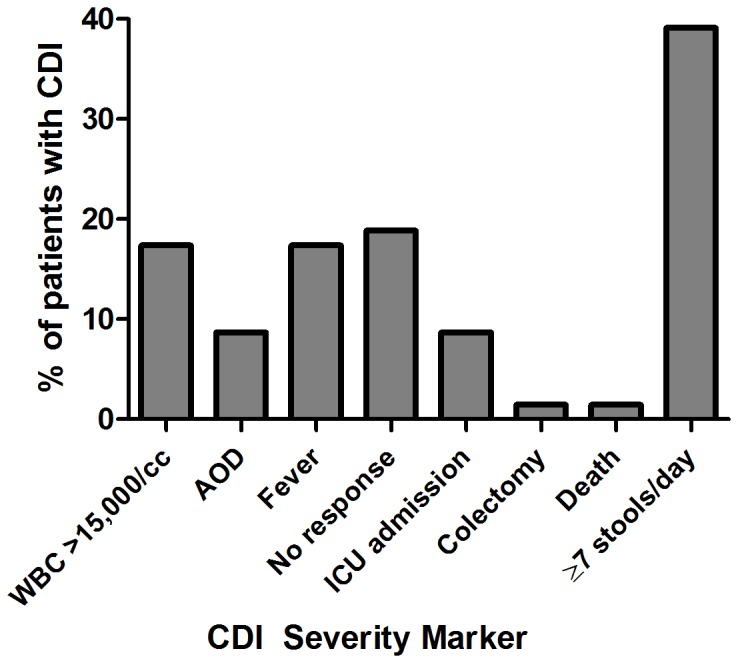
Percent of patients with a CDI severity marker. This bar graph illustrates that having ≥seven stools per day had a much higher prevalence than other severity markers in our cohort. (*WBC*: white blood cell; *AOD*: acute organ dysfunction; *ICU*: intensive care unit).

A total of 69 patients met criteria for inclusion in our study. Median age was 58 (range 20–88) and 37 were female (53%). The median time of PCT measurement was <24 hours after diagnosis and only five patients had received ≥48 hours of treatment for CDI at the time of PCT measurement. Forty-five subjects (65%) had detectable PCT values (range 0.05–140.44). Thirty-four patients were diagnosed based on a positive toxin A/B EIA and 35 based on a positive PCR for toxin B. Twenty-two distinct ribotypes were isolated and eight patients did not have growth on culture (Figure 1). Overall, 21 patients met the criteria for a positive *clinical score*: WBC >15,000 cells/mm^3^ (12 cases), AOD (six cases), fever (12 cases) and 27 had a positive *expanded score*: ICU stay (six cases), death from CDI (one case), no response (13 cases), colectomy (one case). These results are illustrated in Figure 2 and this shows an abundance of severity markers in patients who have elevated PCT values and a scarcity of these markers in patients with low or undetectable PCT values. A total of 27 patients had ≥seven stools per day at the time of enrolment in our study and this high prevalence overshadowed the other severity markers (Figure 3).

### Primary Outcome

By univariate logistic regression, log_10_ PCT was associated with the *clinical score* (OR 3.13, 95% CI 1.69–5.81, *P*<.001) and the *expanded score* (OR 3.33, 95% CI 1.77–6.23, *P*<.001). Age was also associated with the *expanded score* (OR 1.03, 95% CI 1.001–1.06, *P* = .045) but not with the *clinical score*. ECF residence was associated with the *clinical score* (OR 3.5, 95% CI 1.01–12.2, *P* = .049) and the *expanded score* (OR 4.75, 95% CI 1.29–17.5, *P* = .019). Toxin A/B positivity by EIA was associated with the *clinical score* (OR 5.33, 95% CI 1.67–17.04, *P* = .005) and the *expanded score* (OR 5.71, 95% CI 1.95–16.72, *P* = .001). Ribotypes isolated from >five patients (002, 014–020, 027, 053–163, and those with no growth) were assessed for an association with severity. Ribotype 027 showed an association (Table 1) but the other ribotypes did not (data not shown). The following covariates were not associated with either the *clinical score* or the *expanded score*: gender, ADL score, immunosuppression, modified ARC/weighted Charlson-Deyo score, renal disease, concurrent ABI, being on CDI treatment >48 hours, and CDI surveillance definition (Table 1). Furthermore, log_10_ PCT remained a significant predictor of CDI severity after adjusting for each of these factors individually (data not shown). Timing between CDI diagnosis and PCT measurement did not associate with either CDI severity or PCT level (data not shown).

**Table 1:** Odds ratios for predictors of CDI severity scores based on univariate logistic regression.

	*Clinical Score*						*Expanded Score*					
Variable	OR		95% CI		*P*		OR		95%CI		*P*	
log_10_ PCT^1^		3.12		1.69–5.81		<.001		3.33		1.77–6.23		<.001
Positive Toxin A/B EIA^2^		5.33		1.67–17.0		.005		5.71		1.95–16.7		.002
027 Ribotype^2^		7.19		1.27–40.8		.026		11.7		1.32–104		.027
Age^3^		1.02		1.00–1.05		.109		1.03		1.00–1.06		.045
Male Gender^2^		0.82		0.29–2.29		.698		1.12		0.43–2.96		.813
ADL Score^4^		0.99		0.77–1.23		.904		0.98		0.78–1.22		.870
ECF Resident^2^		3.50		1.01–12.1		.049		4.75		1.29–17.5		.019
Charlson-Deyo Score^5^		1.02		0.82–1.27		.869		0.99		0.80–1.22		.920
Modified ARC Score^5^		1.13		0.87–1.48		.367		1.23		0.95–1.60		.112
Immunosuppression^2^		1.14		0.41–3.24		.799		1.01		0.38–2.71		.983
Renal Disease^2^		0.62		0.12–3.25		.569		0.75		0.17–3.29		.703
Treatment >48 hrs^2^		0.55		0.06–5.24		.603		0.37		0.04–3.46		.380
Concurrent ABI^2^		1.20		0.38–3.79		.756		1.35		0.45–4.01		.591
CA^2^		0.98		0.19–3.96		.974		1.04		0.24–4.06		.951
COHA^2^		0.47		0.12–1.53		.234		0.41		0.12–1.24		.130
HOHA^2^		1.30		0.46–3.68		.617		1.67		0.63–4.49		.304

1 ng/mL,

2 true/false,

3 years,

4 range 0–6 for increasing functional impairment,

5 weighted comorbidity index.

**Figure 4:**
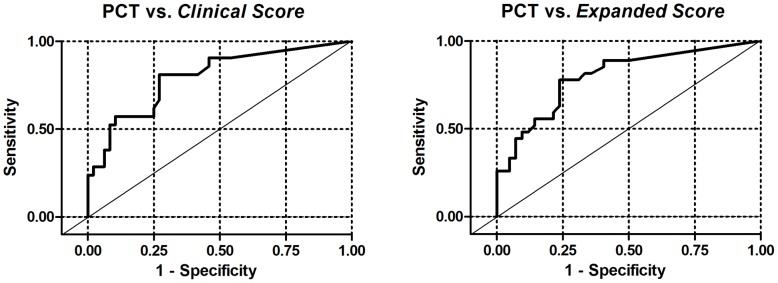
ROC curves of PCT vs. the *Clinical Score* and the *Expanded Score*. The area under the curve for both is 0.8.

The results of the fully adjusted model, which included log_10_ PCT, toxin A/B positivity by EIA, ribotype 027, age, weighted Charlson-Deyo score, CABI, and ECF residence, are shown in Table 2. In the fully adjusted model, log_10_ PCT continued to show a robust association with the *clinical score* (OR 3.09, 95% CI 1.5–6.35, *P* = .002) and the *expanded score* (OR 3.06, 95% CI 1.49–6.26, *P* = .002). Using ROC curve analysis, the optimal cutoff for PCT was determined to be between 0.18 and 0.2 ng/mL with an area under the curve of 0.8 (Figure 4). When used as a diagnostic test, a PCT of >0.2 ng/mL had a sensitivity of 81% and specificity of 72% for the *clinical score* and a sensitivity of 74% and specificity of 76% for the *expanded score*. The negative predictive value of PCT <0.2 for the *clinical score* was 90%.

**Table 2:** Odds ratios for predictors of CDI severity scores based on multiple logistic regression.

	*Clinical Score*						*Expanded Score*						
Variable	OR		95% CI		*P*		OR		95%CI		*P*		
log_10_ PCT^1^		3.09		1.50–6.35		.002		3.06		1.49–6.26		.002	
Positive Toxin A/B EIA^2^		3.11		0.73–13.4		.127		3.56		0.91–14.0		.068	
027 Ribotype^2^		2.66		0.28–25.3		.395		4.71		0.29–77.7		.279	
Age^3^		1.02		0.98–1.06		.441		1.02		0.98–1.06		.316	
Charlson-Deyo Score^4^		0.64		0.38–1.09		.102		0.63		0.37–1.08		.093	
Concurrent ABI^2^		1.34		0.28–6.29		.715		1.41		0.31–6.41		.654	
ECF Resident^2^		1.31		0.23–7.49		.765		1.79		0.28–11.5		.541	

1 ng/mL,

2 true/false,

3 years,

4 weighted comorbidity score.

### Secondary Outcomes

**Figure 5:**
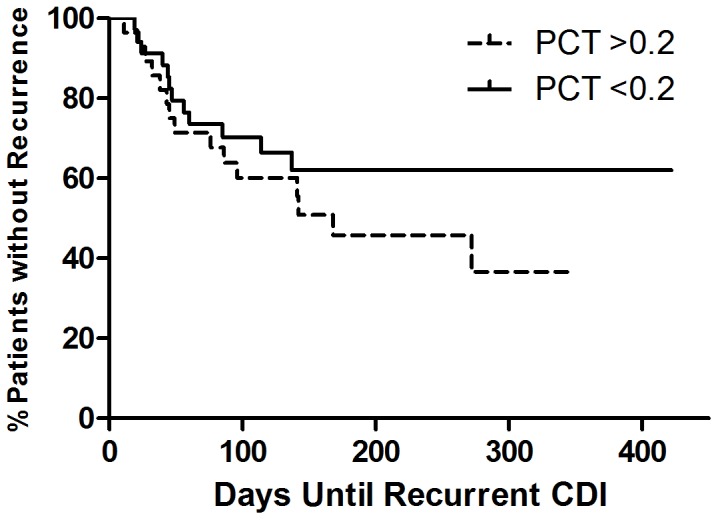
Kaplan-Meier Curve for Recurrence by PCT group. This curve shows a higher recurrence rate for those with PCT >0.2 ng/mL but the hazard rate was not significant (HR 1.57, 95% CI 0.7–3.4, *P* = .246).

Log_10_ PCT did not associate with having ≥seven stools per day and adding this variable to the *clinical score* weakened the association, though it retained significance (OR 1.7, 95% CI 1.1–3.0, *P* = .026). Log_10_ PCT did not associate with length of stay, the need for an increased level of care at discharge, or early relapse (data not shown). Kaplan-Meier analysis (Figure 5) for recurrent disease demonstrated a hazard ratio of 1.57 for those with PCT >0.2 versus those with PCT <0.2 but this did not reach significance (*P* = .246).

## Discussion

Serum PCT is a biomarker with proven clinical utility in bacterial infections (studied in respiratory infections [17], sepsis [18], [19], postoperative infections [20], [21], and ventilator-associated pneumonia [22]) and this study, to our knowledge, is the first systematic investigation to show an association with severity in CDI. This association remained significant when we adjusted for other known risk factors and when we used multiple definitions of severity. Unlike prior studies [13], [29], [30], however, we did not find stool frequency to be a useful severity marker. The high prevalence of having ≥seven stools per day in our cohort can likely account for this difference.

A major finding of our study was that subjects with CDI and a PCT of <0.2 ng/mL were unlikely to have clinical measures of severe infection or an adverse CDI-related outcome, as evidenced by the negative predictive value of 90% for the *clinical score*. This suggests that patients with a low PCT can be managed more conservatively than those with higher values, who might require more aggressive therapy, hospital admission, or closer observation. The choice to use oral metronidazole, for example, which is recommended for mild-moderate CDI (WBC <15,000 cells/mm^3^ or serum creatinine level less than 1.5 times the premorbid level) [10], might be supported by a low PCT value but avoided if a patient has a high PCT.

Our study measured PCT on the same day or shortly after diagnosis when many patients had already developed other severity markers. Due to our study design, we were unable to determine whether PCT levels at the onset of symptoms similarly predict disease severity or outcome. Although there existed some variability in the time between CDI diagnosis and PCT measurement, an association between timing and either severity or PCT value was not observed. Future studies will be needed to validate our results, to extend them to understand whether PCT levels measured at the earliest stages of infection predict severe disease, and to define the role for biomarkers such as PCT in treatment algorithms for CDI.

Though we hypothesized that high PCT levels would associate with an elevated risk of recurrence, in our study this did not reach significance. Although initial disease severity has been associated with recurrent disease [31], the lack of association in our study could be related to the small cohort size. If elevated PCT is found to be associated with disease recurrence in an appropriately designed study, treatment algorithms can be adjusted to maximize resource allocation. An example would be to use fidaxomicin in place of vancomycin for initial therapy, as it has been shown to be more effective in reducing recurrent disease [32]. Additionally, measurements in patients during suspected recurrent disease need to be evaluated to assess if PCT may help with diagnosis and management in this patient group.

The clinical significance of diagnosing CDI based upon toxin EIA versus PCR is unclear [33], [34]. The possibility that the detection of toxin A/B by EIA more robustly predicts disease severity than toxin gene detection by PCR is an intriguing concept. In fact, we observed this in univariate analysis but not in the final model. This relationship deserves further study. Our hospital’s practice of using Cary-Blair transport medium for stool samples may have affected our results by diluting stool toxins to below detectable limits.

A significant amount of ribotype diversity was identified among the isolates in this study (Figure 1), suggesting that a particular strain was not driving severity in our cohort. Though ribotype 027 was associated with severity on univariate analysis, this was not seen in the final model. This lack of association between the so-called “hypervirulent” ribotype 027 [35], [36] and CDI severity has been observed previously and remains controversial [25], [37], [38], [39], [40], [41].

This study was limited by including a relatively small number of patients while looking at multiple clinical severity markers. Given this, we used binary variables as opposed to continuous ones to explore the association with PCT and severity in CDI. While we were able to establish an association with the presence or absence of clinically severe CDI, we were unable to discriminate across degrees of severity the way a continuous score would. Some of the PCT measurements occurred more than 24 hours after diagnosis and while on effective therapy. Although this was only true for 7% of our patients, this limited our ability to interpret PCT’s temporal relationship to CDI severity markers and possibly diluted the association for those patients on therapy. Although we attempted to account for it, more data are needed before we can comment on the validity of PCT measurements for CDI severity in patients with CABI’s. Finally, the stability of PCT in frozen samples could have affected our results, but prior data and the information in the bioMérieux package insert suggest good stability over time [42], [43].

In summary, this study sheds new light on the potential utility for serum PCT measurements in persons with CDI. Although further study in a larger cohort is required to validate and to better explore the relationship, PCT does show some promise as a biomarker for CDI severity.
